# Comparison of atomic scale dynamics for the middle and late transition metal nanocatalysts

**DOI:** 10.1038/s41467-018-05831-z

**Published:** 2018-08-23

**Authors:** Kecheng Cao, Thilo Zoberbier, Johannes Biskupek, Akos Botos, Robert L. McSweeney, Abdullah Kurtoglu, Craig T. Stoppiello, Alexander V. Markevich, Elena Besley, Thomas W. Chamberlain, Ute Kaiser, Andrei N. Khlobystov

**Affiliations:** 1 Electron Microscopy of Materials Science, Central Facility for Electron Microscopy, Ulm University, Albert-Einstein-Allee 11, Ulm, 89081 Germany; 2 School of Chemistry, University of Nottingham, University Park, Nottingham, NG7 2RD United Kingdom; 3 Institute of Process Research and Development, School of Chemistry, University of Leeds, Leeds, LS2 9JT United Kingdom

## Abstract

Catalysis of chemical reactions by nanosized clusters of transition metals holds the key to the provision of sustainable energy and materials. However, the atomistic behaviour of nanocatalysts still remains largely unknown due to uncertainties associated with the highly labile metal nanoclusters changing their structure during the reaction. In this study, we reveal and explore reactions of nm-sized clusters of 14 technologically important metals in carbon nano test tubes using time-series imaging by atomically-resolved transmission electron microscopy (TEM), employing the electron beam simultaneously as an imaging tool and stimulus of the reactions. Defect formation in nanotubes and growth of new structures promoted by metal nanoclusters enable the ranking of the different metals both in order of their bonding with carbon and their catalytic activity, showing significant variation across the Periodic Table of Elements. Metal nanoclusters exhibit complex dynamics shedding light on atomistic workings of nanocatalysts, with key features mirroring heterogeneous catalysis.

## Abstract (web summary)

The atomistic behaviour of nanocatalysts still remains largely unknown. Here, the authors reveal and explore reactions of nm-sized clusters of 14 technologically important metals in carbon nano test tubes using time-series imaging by atomically-resolved transmission electron microscopy.

## Introduction

Tiny clusters of metal nanocatalysts combine numerous benefits of homogeneous and heterogeneous catalysts^1^, but they also pose a fundamental challenge related to the exact nature of the catalytically active centres which remain poorly defined during the reaction. Assuming that all nanoclusters are identical at the start of the reaction (in an ideal case), their shapes and sizes will undergo continuous changes due to re-faceting^2^ and Ostwald ripening processes, which in turn have unpredictable impacts on the activity and selectivity of the nanocatalyst. Clearly, the ever-changing nature of the metal nanoclusters during the reaction creates a significant obstacle to the rational design of new nanocatalysts, with the only way to assess the relationship between the atomic structure of the nanocluster and its catalytic activity being imaging simultaneously the dynamic transformations of the metal nanocluster and the rate and selectivity of the promoted chemical reactions. Transmission electron microscopy (TEM) nowadays offers atomic spatial and high temporal resolution, inaccessible in other microscopy methods. With the help of advanced environmental TEM techniques, the in-situ observation of nanocatalysis with atomic resolution can be achieved, but the influence of the e-beam, which has proven to have a dramatic effect on the dynamics and performance of nanocatalysts, has been largely neglected^3–5^.

In this study, the structural dynamics and reactions of nanometre-sized clusters of 14 technologically important metals in carbon nano test tubes are investigated and compared by employing the electron beam in TEM simultaneously as an imaging tool and stimulus of the reactions which shows nanoclusters in action and provides a new guide for the development of better catalysts for important processes involving carbon–carbon (C–C) bond dissociation and formation.

## Results

### Dynamics of metal nanoclusters under e-beam irradiation

**Fig. 1:**
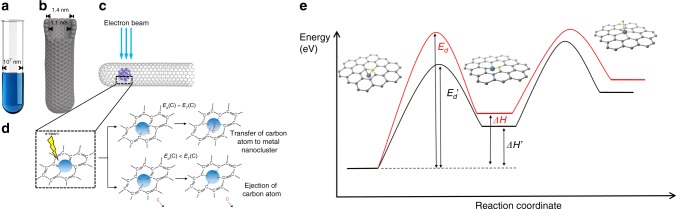
Fundamental principles of ChemTEM. In comparison to conventional glass test tubes (**a**), which have a diameter of ${\scriptstyle\sim}$1 cm (10^7^ nm), carbon nanotubes (**b**), possess a channel with a van der Waals diameter of 1.1 nm making them a suitable vessel for the entrapment of nano-sized samples of different chemical elements, that can also act as an effective nanoreactor provided that the nanotube structure is not defective and its inner channel is free of impurities. Our methodology enables the insertion of clusters of 20–60 metal atoms within nano test tubes (**c**) and investigation by aberration corrected high resolution TEM (AC-HRTEM) reveals the nanoclusters’ structures and chemical transformations at the atomic scale. The electron beam serves a dual purpose: some electrons (a tiny fraction) transfer a portion of their kinetic energy to the metal and carbon atoms, as described by the equation for *E*_*T*_ above, which activates chemical transformations in the sample, whilst most of the electrons are transmitted through or scattered by the specimen generating two-dimensional images, the collation of which into time series enables the results of the induced transformations to be observed over time. **d** The presence of metal nanoclusters reduces the energy barrier for the displacement of a carbon atom (*E*_*d*_) from the nanotube which can be either ejected, leading to a vacancy defect (bottom diagram) or converted into another carbon structure (top diagram), with both processes facilitated by the metal nanocluster (Supplementary Fig. 9). **e** A schematic energy profile of the formation of a vacancy defect illustrating a link between *E*_*d*_ and the enthalpy change (*ΔH*): the formation of metal-carbon (M–C) bonds between the metal nanocluster (represented by a single metal atom) and the dangling bonds of the defect make the defect more stable, thus lowering the energy threshold for defect formation (black curve) as compared to the same process in the absence of a metal nanocluster (red curve)

Here we utilise carbon nanotubes (NTs) with an internal diameter of ca. 1.4 nm for entrapment of tiny samples, in the form of 20–60 atom clusters of 14 technologically important metals from a central part of the periodic table of elements (Fig. 1a–c, Supplementary Table 1, Supplementary Figs. 1–4). This experimental strategy for investigation and comparison of metal nanocluster behaviour builds on our previously reported concept of ChemTEM, in which the electron beam in the TEM simultaneously triggers, controls and images the result of chemical reactions^6^. Importantly, the rates of the reactions are directly proportional to the dose rate of the e-beam, readily controlled by the TEM settings, which can therefore be tuned to capture time series images of the results of individual reactions. These images can then be combined together to form a stop-frame movie bearing information on reaction dynamics and mechanisms at the atomic level^7^. In ChemTEM the reactions are driven by isolated events in which direct momentum transfer from a fast incident electron to an atom results in a shift of the atom from its equilibrium position, potentially leading to bond dissociation. The exact amount of kinetic energy transferred from the e-beam to the atom is described as

1 $$E_T = \frac{{2m_nE(E + 2m_ec^2)}}{{(m_n + m_e)^2c^2 + 2m_nE}}\sin^2\left( {\frac{\theta }{2}} \right) = E_{T\_{\rm max}}\sin^2\left( {\frac{\theta }{2}} \right)$$

where *m*_*n*_ = mass of atoms, *m*_*e*_ = mass of electron, *E* = energy of the e-beam, *c* = speed of light, and *θ* *=* the electron scattering angle.

**Fig. 2:**
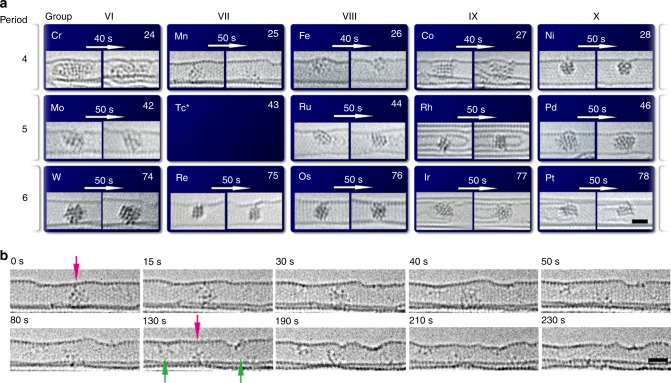
Dynamic behaviour of 14 metal nanoclusters under e-beam irradiation. **a** Typical examples of 80 keV AC-HRTEM images of 14 transition metal nanoclusters in carbon nanotubes, showing their structural transformations after 40-50 s of electron irradiation. **b** Time-series images of a Mn nanocrystal breaking down to individual atoms under the electron beam. The intact Mn nanocrystal (indicated by a magenta arrow in the 0 s frame) gradually breaks up into smaller clusters (indicated by green arrows in the 130 s frame) and single atoms during continuous irradiation for 230 s. *Tc is a rare radioactive element, which was not studied in this work. The scale bar is 1 nm

Under the 80 keV e-beam in our experiments, the maximum kinetic energy transferred to transition metals, *E*_*T_*max_(M) (Eq. 1), is in the range of 1–4 eV; *E*_*T_*max_(M) decreases slowly across a period (for example, from 3.64 eV for Cr to 3.23 eV for Ni in period 4) but decreases rapidly down a group (for example, from 3.23 eV for Ni, to 1.78 eV for Pd and 0.97 eV for Pt) (Table 1). During a 40–50 s period of e-beam irradiation (corresponding to a cumulative dose of ~4 × 10^7^ e^−^ nm^−2^ to 5 × 10^7^ e^−^ nm^−2^) initially well-ordered nanoclusters of metals undergo transformations to different extents (Fig. 2a). For example, Mn nanoclusters show the most dynamic behaviour among the 14 metals (Fig. 2b and Supplementary Movie 1): a compact crystalline Mn nanocluster becomes disordered after 15 s of electron irradiation (dose rate 1 × 10^6^ e^−^ nm^−2^ s^−1^), and subsequently breaks down to smaller clusters (40–190 s) and then all the way to single atoms (210–230 s). Under the same conditions, Cr and Co nanoclusters exhibit similar processes of disordering into an amorphous, liquid-like state, but do not dissociate completely like Mn nanoclusters (Supplementary Fig. 5). In contrast, all other metal nanoclusters are less labile under the e-beam, changing their shapes continually but maintaining their crystalline features. This indicates that the *E*_*T*_ for these metals is sufficient to perturb atomic positions, but not to displace metal atoms from the nanocluster irreversibly (Supplementary Figs. 5, 6).

**Table 1:** Comparison of energy transferred to metal with strength of metallic bonding

Metal	_24_Cr	_25_Mn	_26_Fe	_27_Co	_28_Ni	_42_Mo	_43_Tc	_44_Ru	_45_Rh	_46_Pd	_74_W	_75_Re	_76_Os	_77_Ir	_78_Pt
*E*_coh_ (eV)	4.10	2.92	4.28	4.39	4.44	6.82	6.85	6.74	5.75	3.89	8.90	8.03	8.17	6.94	5.84
*E*_*T_*max_ (eV)	3.64	3.45	3.39	3.21	3.23	1.97	1.93	1.87	1.84	1.78	1.03	1.02	1.00	0.99	0.97
[*E*_*T_*max_/*E*_coh_] × 100 (%)	89	118	79	73	72	29	28	28	32	46	12	13	12	14	17

Cohesive energy of metals (*E*_coh_) is compared to maximum transferred energy (*E*_*T_*max_) from the 80 keV e-beam for different transition metal atoms

Within the framework of our ChemTEM studies, the e-beam delivers an exact and controlled amount of energy to the atoms, causing chemical reactions if the maximum kinetic energy delivered from an incident electron, *E*_*T_*max_ (*θ* *=* 180°), exceeds the threshold energy for the particular chemical reaction. In the case of the metal (M) nanoclusters, dissociation of the M–M bond is required for an irreversible displacement of the atom from its original position, which means that the observed dynamics of the particular metal nanocluster is directly proportional to its *E*_*T_*max_ (defined by the energy of the e-beam and the atomic weight; Eq. 1) and inversely proportional to the strength of the interatomic M–M bonding present (which correlates with the basic physicochemical properties of the metals, such as their cohesive energy, *E*_coh_). Assuming that bulk *E*_coh_ scales down with nanocluster size similarly for all 14 metals, comparison between the ratios of the energies (*E*_*T_*max_/*E*_*coh*_) for each metal can serve as an approximate guide for the dynamics of different metal nanoclusters of similar sizes under the e-beam (Table 1). The bulk cohesive energies for the 14 metals studied range from 2.9 eV (for Mn) to 8.9 eV (for W) and remain greater than the corresponding maximum transferred energy *E*_*T_*max_ (0.97–3.64 eV) in all cases except for Mn (Table 1). Thus, we expect that the Mn nanocluster will be affected to the largest extent by the 80 keV e-beam, which is consistent with our experimental observations. Furthermore, we expect metal nanoclusters such as Cr, Fe, Co and Ni with high *E*_*T_*max_/*E*_coh_ ratios to be significantly perturbed by the 80 keV e-beam but to a lesser extent than Mn. All other metals with significantly lower *E*_*T_*max_/*E*_coh_ ratios we expect to be significantly less dynamic. This simple analysis is in line with all of our experimental observations (Fig. 2a) with two notable exceptions, Fe and Ni, which exhibit less dynamic behaviour than expected (Supplementary Fig. 6). This comparative analysis shows that the observed trends cannot be explained solely on the basis of the properties of the metals, and therefore interactions between the metal and the surrounding nanotube must also be considered. In addition, the e-beam stimulated quasi-melting process is compared with thermally stimulated melting processes in Supplementary Discussion 1 and Supplementary Table 2, highlighting the similarities and differences observed in the two processes.

### Bonding of metal nanoclusters with carbon

**Fig. 3:**
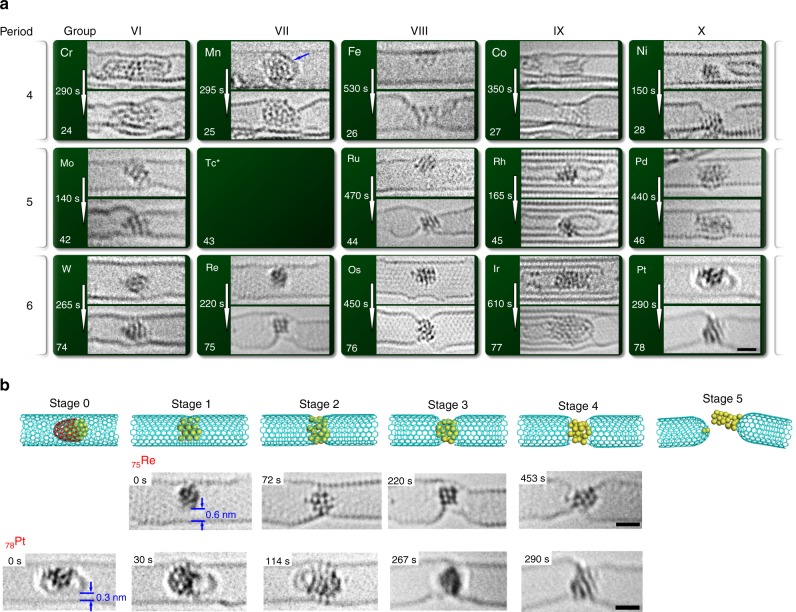
Bonding of 14 metal nanoclusters with carbon. **a** Representative examples of 80 keV AC-HRTEM images of 14 transition metal nanoclusters in carbon nanotubes, highlighting their interactions and reactions with the carbon nanotubes. The carbon shell covering Mn which stabilises the Mn nanocluster against impact of the e-beam is marked by a blue arrow. **b** Schematic diagrams of the characteristic stages of defect formation and propagation in the host NT and time series AC-HRTEM images of Re and Pt, respectively, illustrating the evolution of the nanoclusters and NTs. The gap which is initially observed between the nanocluster and the bottom wall of the nanotube (≥0.3 nm) indicates that no covalent bonding is present between the metal and carbon along this direction at the start of the process, with the extent and rate of subsequent nanotube transformation related to the chemical nature of the metal. The scale bar is 1 nm

Carbon atoms—due to their low atomic weight—receive significantly more kinetic energy from the 80 keV electrons (*E*_*T_*max_(C) *=* 15.8 eV; Eq. 1) than the atoms of any of the transition metals. However, because of the strong interatomic bonding present between the carbon atoms in a pristine nanotube, the transferred energy remains below the displacement threshold (*E*_*d*_(C) ≈ 17 eV)^8^, such that—in the absence of metal nanoclusters—defect-free carbon NTs do not show any significant transformations in 80 keV AC-HRTEM experiments. The metal nanoclusters encapsulated in the nano test tubes in our experiments are in direct contact with the carbon atoms and appear to activate defect formation (Fig. 3a). Surprisingly, the extent and rates of NT defect formation in our measurements vary widely from metal to metal. Over 120,000 meticulously measured TEM images of transition metal nanoclusters (diameter < 2 nm) encapsulated in NTs were recorded in time series using AC-HRTEM (80 keV accelerating voltage, at a dose rate of ~10^6^ e^−^ nm^−2^ s^−1^). Detailed analysis of the resultant time series reveals a variety of interactions between the metal nanoclusters and the carbon nanotube which we classify into five distinct stages of a common sequence. (Fig. 3b). The extent of the interactions, i.e. the specific stage that a particular metal reaches, and the rate at which this sequence of behaviour occurs, i.e. the time taken in seconds to get to each stage, varies between metals and can be used to provide vital, semi-quantitative data on the chemical nature of the 14 different metal nanoclusters. Stage 1 corresponds to the formation of an initial defect in the nanotube sidewall due to the loss of a small number (between 1 and 4) of carbon atoms and bonding of the metal nanocluster to the defect, importantly at this stage the metal nanocluster remains separated from the rest of the nanotube by a distinct, 0.3 nm sized van der Waals gap. This stage is a common starting point present at the beginning of the observations for all metals (except Mn and Pt), and is followed for the majority of metals by stage 2 in which the metal nanocluster is bonded to the entire cross section of the nanotube channel due to extensive loss of carbon atoms. There is a significant spread of time required for different metals to reach stage 2 (Table 2), the distinctive feature of this stage being the reduction of the gap between the metal nanocluster and the concave surface of the nanotube to distances smaller than 0.3 nm. Several metals then proceed to stage 3, manifested as a sharp contraction of the nanotube cross section around the metal nanocluster and partial protrusion of metal atoms through the nanotube sidewall due to further loss of carbon atoms. In stage 4 almost all carbon atoms around the metal nanocluster are removed, such that the nanocluster is exposed, held in place only by the tips of the two severed sections of the NT, which eventually dissociate from the metal leading to a complete breakdown of the nano test tube in the final stage (stage 5). Among the 14 metals investigated, Fe and Ni nanoclusters are most active towards defect formation in the nanotube, as they reach stage 5, rupturing the NTs during AC-HRTEM imaging (Supplementary Fig. 7). The observed behaviour patterns, as well as the rates for each metal, are reproducible from nanotube to nanotube and from area to area, as long as the size of the metal nanocluster remains approximately the same (limited to ~1–2 nm by the NT diameter). The levels of reproducibility of these AC-HRTEM measurements are demonstrated in the Supplementary Information (Supplementary Discussion 2 and Supplementary Fig. 8). Overall, ChemTEM measurements enable discrimination of the activities of transition metals towards defect formation by comparing the stages the metals reach and the time required:

**Table 2:** Activity of metal towards defect formation in nanotube sidewall

	Pd	Rh	Ir	Cr	W	Mo	Ru	Co	Mn	Re	Fe	Os	Pt	Ni
Stage 0	–	–	–	–	–	–	–	–	0 s	–	–	–	0 s	-
Stage 1	0 - 440 s	0 – 165 s	0 s	0 s	0 s	0 s	0 s	0 s	73 s	0 s	0 s	0 s	30 s	0 s
Stage 2	–	–	610 s	290 s	265 s	140 s	233 s	207 s	98 s	72 s	328 s	157 s	114 s	28 s
Stage 3	–	–	–	–	–	–	470 s	350 s	295 s	220 s	430 s	375 s	267 s	72 s
Stage 4	–	–	–	–	–	–	–	–	–	453 s	530 s	450 s	290 s	150 s
Stage 5	–	–	–	–	–	–	–	–	–	–	650 s	545 s	–	158 s

Time required to reach the five characteristic stages of defect formation and propagation is compared for the 14 transition metal nanoclusters. The time reported can be used for semi-quantitative comparison of the ability of the different metals to promote defect formation in the host NT and related to the nature of the nanocluster-carbon bonding present for each metal. The times quoted (with an experimental measurements error of ±0.5–2.0 s due to variations in the image exposure time) are measured from the time series of AC-HRTEM images for the nanoclusters shown in Fig. 3a. The detailed corresponding time-series AC-HRTEM images of the Table 2 are shown in Supplementary Fig. 7

Ni > Pt > Os > Fe ≫ Re > Mn ≫ Co > Ru ≫ Mo > W > Cr > Ir ≫ Rh > Pd (Series 1)

For a defect to emerge in the NT sidewall, the C–C bonds must dissociate first, followed by the formation of M–C bonds between the metal nanocluster and the dangling bonds of the nanotube (Fig. 1d). The metal nanocluster thus plays a dual role in this process: (*i*) reducing the energy threshold for carbon atom displacement (by at least 1.2 eV to ensure *E*_*T_*max_(C) > *E’*_*d*_(C); Fig. 1e) and (*ii*) stabilising the vacancy defect via M–C bonding (in the presence of metal, the enthalpy of defect formation is less endothermic due to stabilisation of the dangling bonds |*ΔH*^’^| < |*ΔH*|; Fig. 1e). For example, *ΔH’*(Ru) is lower than *ΔH* without Ru by ~6.5 eV (Supplementary Fig. 9). While the latter appears to be more significant, both roles are linked by the Bell-Evans-Polanyi principle—relating the activation energy to the enthalpy for reactions of similar type— which, if applied to our ChemTEM study, provides a working relationship between the energy barrier *E*_*d*_*’* and the enthalpy *ΔH’* for each particular metal (e.g. the lower *ΔH’* —the lower *E*_*d*_*’*; Fig. 1e), which is ultimately defined by the bonding of the metal nanocluster with carbon. Previously, strong bonding of the transition metal atoms with mono-vacancy^9,10^ and di-vacancy defects^11,12^ in the graphitic lattice has been demonstrated theoretically and experimentally for isolated cases of transition metals, indicating that the presence of metal can facilitate formation of defects under e-beam irradiation^13–15^. Our own ChemTEM studies previously limited to individual metals (Ni^16,17^, Re^18^) or metal triads (W-Re-Os^19,20^, Fe-Ru-Os^21^) have indicated a strong correlation between the observed dynamics of defect formation in carbon structures and the position of the metal in the period or group^19,20^. Therefore, this evidence hints that the different rates of NT defect formation promoted by different metal nanoclusters are linked to the relative bonding energies of those nanoclusters with carbon. However, in order to demonstrate this, a global comparison for the transition metals, revealing fundamental trends across the periodic table is required.

The metal-promoted C–C bond dissociation and M–C bond formation events taking place in the ChemTEM experiments are crucially important elementary steps in many industrial reactions, including the cracking and reforming of hydrocarbons, processes which underpin the global petrochemical industry. Modern organometallic chemistry is not yet able to provide an overarching understanding of the M–C bonding ability across the periodic table, but it gives glimpses of the trends within a given group, for example, indicating that heavier transition metals should be expected to form stronger M–C bonds^21^. However, our measurements provide a global view of transition metals’ interaction with carbon and demonstrate no significant correlation between its efficiency and the metal’s position in the periodic table. This suggests a complex interplay of the element-specific valence electron configuration, electronegativity and the size of the atom—as discussed recently in comparative theoretical studies of transition metals on carbon^9–12^, that cannot provide a simple rule of thumb for explaining or predicting the semi-quantitative trends obtained from our experiments.

### Catalytic activities of metal nanoclusters

**Fig. 4:**
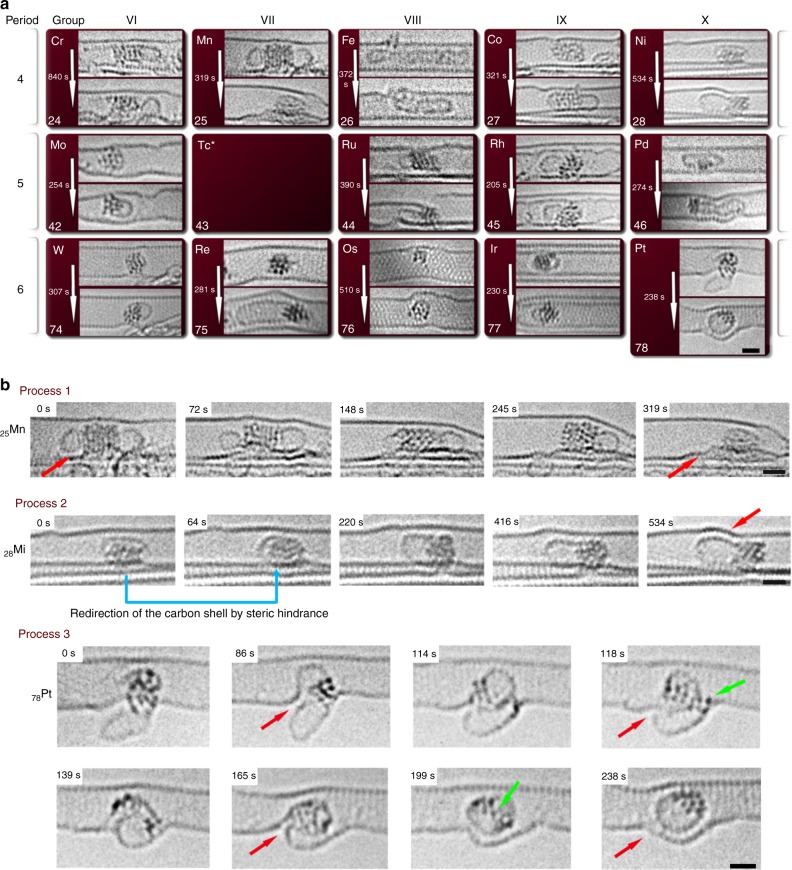
Catalysis by 14 metal nanoclusters. **a** Representative examples of 80 keV AC-HRTEM images of the 14 transition metal nanoclusters in carbon nanotubes highlighting different examples of catalytic behaviour that are manifested as the rearrangement and formation of a variety of carbon nanostructures. **b** Time-series AC-HRTEM images illustrating three fundamental types of metal nanocluster activities. ‘Process 1′: a Mn nanocluster promotes incorporation of the native carbon shell into the nanotube sidewall, which causes the lower part of the NT to warp, as indicated by the red arrows. ‘Process 2′: a Ni nanocluster abstracts carbon atoms from a point of contact with the host NT and promotes the formation of a new carbon structure. The direction in which the carbon shell grows is dictated by steric hindrance imposed by the host nanotube (indicated with blue arrows). The loss of carbon atoms from the host NTs leads to structural deformation (indicated with red arrow). ‘Process 3′ (a combination of ‘Process 1′ and ‘Process 2′): a Pt nanocluster abstracts carbon atoms from the host NT and restructures them into a carbon shell and simultaneously promotes incorporation of the native carbon shell into the host NT (areas of new carbon structure formation and sealing of a loop-and-hole structure are indicated by red arrows, individual Pt atoms dissociated from the nanocluster are indicated by green arrows). The scale bar is 1 nm

In addition to defect formation promoted by the metals in NTs, the nanoscale clusters facilitate rearrangement of one form of carbon into another (Fig. 4a). There are three typical transformations (Fig. 4b), which we observe at the atomic level: process 1 is described as a transfer and incorporation of native carbon nanostructures (Supplementary Fig. 10) from the metal nanoclusters into the carbon nanotube sidewall, such as in the case of the Mn nanocluster with two protruding carbon shells (Fig. 4b) that become incorporated into the nanotube under ChemTEM conditions, the first shell within 148 s, and the second within 171 s, resulting in deformation of the lower NT wall. Cr and Fe also exhibit process 1 type behaviour but to a lesser extent compared to Mn. In contrast, process 2 can be exemplified by Ni nanocluster, which facilitates the transformation of carbon in the opposite direction to process 1 by extracting carbon atoms from the nanotube sidewall and transforming them into a new carbon shell. The Ni nanocluster, ~1 nm in size, can be observed interacting with the host NT and abstracting carbon atoms from it, thus catalysing the growth of the carbon structure to a length of 1.18 nm in 534 s (Supplementary Movie 3). The direction of the growth of the carbon structure is dictated by steric confinement imposed by the host-nanotube that can be clearly observed (indicated with blue arrow, Fig. 4b). The host NT undergoes deformation due to the loss of carbon atoms as a result of process 2 (indicated with red arrow, Fig. 4b). Indeed, AC-HRTEM observations for all transition metals were found to be reproducible, such that under the same conditions (e.g. same nanocluster size, same e-beam energy and dose rate) behaviour of a particular metal can be reproduced from area to area, and from nanotube to nanotube as demonstrated in the Supplementary Discussion 2 and Supplementary Fig. 8. Re, Os, W and Co nanoclusters exhibit process 2 type behaviour, similar to Ni, while Re nanoclusters show the same process but at a much higher rate, forming a long single-walled NT. Mo, Ru and Pd nanoclusters also catalyse process 2 type transformations but by transferring carbon atoms from the original NT to pre-existing carbon shells rather than creating new ones. Distinct from both processes is the unique hybrid process, process 3 (Fig. 4b and Supplementary Movie 4) which is a combination of processes 1 and 2: the Pt nanocluster protruding halfway through a defect in the NT sidewall catalyses the simultaneous transformation of carbon from the nanotube to a shell inside the NT and incorporation of the outer shell into the NT, resulting in a loop-and-hole structure after 86 s (indicated by a red arrow, Fig. 4b). The loop-and-hole structure changes in shape (between 118 s and 162 s) and becomes completely sealed off at 199 s. Finally, at 238 s, the Pt is incorporated within an internal carbon shell, while a nano-protrusion is formed on the host NT.

Importantly, in our experiments we can directly visualise changes in the atomic structure of the metal nanocluster concurrently with carbon structure growth, thus providing spatial and temporal resolution unparalleled by any other method by virtue of the momentum transferred directly from the incident electrons to the atoms. In general, most of the 14 transition metal nanoclusters are more dynamic when they begin bonding to carbon and facilitating rearrangements in the NT structure. For example, the W and Ni nanoclusters continually and rapidly change their structures as the new carbon structures grow from their surface (Fig. 4b, Supplementary Fig. 8, Supplementary Movies 2, 3). This strikingly illustrates the key challenge of nanocatalysis—metal nanoclusters changing their atomic structures during the reaction, that is very similar to the behaviour predicted theoretically^22,23^. Indeed, it is the metastable and ever-changing nature of metal nanoclusters that precludes chirality-selective growth of NTs. Our results may guide the search for conditions of chirality-selective growth of NTs that could be realised by utilising structurally stable alloy catalysts^24^. In addition, the experimental conditions are milder (room temperature, 80 keV e-beam) to the previous studies on in-situ growth of carbon NTs in environmental TEM on particles of catalyst^25–29^, yet the activity of the small W, Ni and Re nanoclusters appear to be sufficiently high to promote the growth of carbon structures, which indicates the influence of e-beam in environmental TEM experiments cannot be neglected. Furthermore, the presented ChemTEM observations provide direct evidence of dynamic single-atom catalysis, which is predicted theoretically:^30^ for example, in the arresting behaviour of the Pt nanocluster in process 3, the nanocluster acts as a viscous liquid dispersing to individual atoms (Fig. 4b, 118 s, marked with the green arrow) and then re-clustering over time (Fig. 4b, 238 s). Such individual atoms are expected to co-exist with nanocatalysts and play an important role owing to their high surface energy^31^, In all above examples, we directly demonstrate the highly dynamic nature of metal nanoclusters during reactions, which could now be harnessed to tune and improve the performance of nanocatalysts^32^. In addition, residing in carbon NTs, the nanoclusters of all 14 metals did not exhibit any significant Ostwald ripening or coalescence, both factors that usually plague the performance of these materials in practical applications. This strategy of utilising a confined environment to stabilise metal nanoclusters could therefore be utilised in the development of more durable and re-usable nanocatalysts in the future.

As all of the measurements were carried out under the same conditions (same energy and dose rate of the electron beam, temperature, pressure) the abilities of the different metals to promote transformations in carbon NTs can be directly and semi-quantitatively compared, thus shedding light on their relative catalytic activities. Expressed in terms of the length change (the changes in surface area and in the number of carbon atoms are estimated in Supplementary Table 3) in the carbon structure promoted by a particular metal (e.g. size change over time, Table 3), the observed catalytic activities appear in the following order:

**Table 3:** Catalytic activity of metals

	Rh	Ir	Cr	Os	W	Mo	Ru	Pd	Co	Ni	Fe	Mn	Re	Pt
*Δl* (nm)	0	0	−0.10	+0.15	+0.45	+0.48	+0.57	+0.68	+0.90	+1.18	− 2.00	− 2.10	+2.18	+2.29

Comparison of the total length changes of the formed/consumed carbon structures. Comparison of the total length changes of the formed/consumed (+/−) carbon structures is considered as relative ability of the 14 metal nanoclusters to promote changes in nanotubes and is used for semi-quantitative comparison of their catalytic activities

Pt > Re > Mn > Fe ≫ Ni > Co > Pd > Ru > Mo > W > Os > Cr > Ir = Rh (Series 2)

As well as being highly dynamic, Pt and Re clearly exhibit the highest activity among the metal nanoclusters, which is consistent with the significant role these metals have in industrial synthesis, such as the Pt-Re catalysts that are widely used in catalytic reforming and other reactions involving transformations of C-C bonds.

## Discussion

**Fig. 5:**
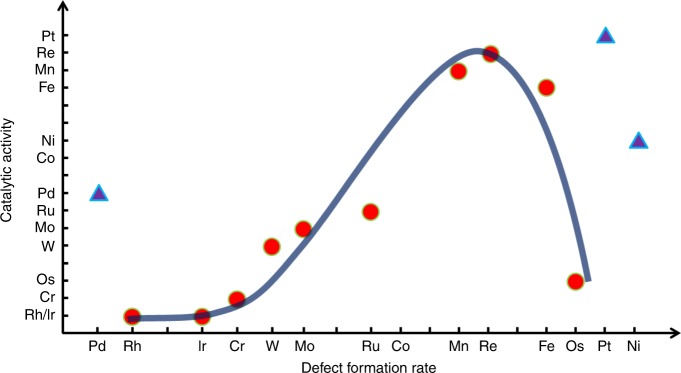
Comparison of 14 transition metals. The level of catalytic activity observed for metal nanoclusters by ChemTEM imaging at 80 keV (series 2 in text) plotted against the rate of defect formation in nanotubes promoted by metal nanoclusters (series 1 in text, linked to nanocluster-carbon bonding) under the same conditions, providing a qualitative link between the two trends. A clear volcano-shape correlation is observed reflecting the Sabatier principle of heterogeneous catalysis for all metals, except those of group 10 (Ni, Pd and Pt)

If, as suggested above, the activity of metal nanoclusters towards defect formation in NTs is indeed related to the bonding energy between the nanocluster and carbon, series 1 should correlate with the catalytic activities of the different metals, series 2, as a volcano-shaped plot—a graphical representation of the Sabatier principle that defines an effective catalytic surface to be neither too reactive nor too inert^33^, so that it both binds effectively to the reactants and readily releases the products of the reaction. The Sabatier principle has become firmly established in the foundations of heterogeneous catalysis^34^, guiding the discovery of new catalysts^35^, including those for carbon nanostructure growth^36^. However, because of the highly dynamic surface of metal nanoclusters, there is no certainty that the same principle would apply for nanocatalysis. Our results for the 14 transition metals show a striking correlation of series 1 with series 2 with the classic volcano shape (Fig. 5), thus not only verifying that series 1 is connected to the relative nanocluster-carbon bonding energies, but also suggesting that the mode of action of nanocatalysts, even ones that are tiny in size (20–60 atoms), is closer to that of heterogeneous rather than homogeneous catalysts.

Three metals (Ni, Pd and Pt) significantly deviate from the general trend, exhibiting higher catalytic activities than should be expected solely from consideration of the Sabatier principle. It is significant that the three deviating metals belong to the same group, group 10, and possess the highest number of *d*-electrons amongst the metals we studied. The filling of *d*-orbitals along a period of transition elements has several important consequences, including a sharp decrease in the contribution of the *d*-orbitals to metallic bonding for Ni, Pd and Pt as compared to other metals^37^. Furthermore, the high occupancy of the *d*-orbitals in group 10 metals allows only transient interactions with surrounding carbon and high diffusivity of carbon atoms through the metal nanocluster, ensuring that no well-defined interface can exist between these metal nanoclusters and carbon structures under demonstrated ChemTEM conditions, thus explaining why the group 10 metals appear to disobey the Sabatier principle.

For over six centuries scientists have been using laboratory glassware (test tubes, retorts, flasks, beakers etc.) to discover and investigate the properties of chemical elements and the mechanisms of chemical reactions. These traditional methods work well for reactions where the key parameters stay the same, but in nanocatalysis owing to the largely unpredictable and dynamic behaviour of the active centres, new approaches are required. Recent advancements in TEM together with the use of nano test tubes offer a radically new way to study and compare chemical processes at the atomic level^38^. Our use of a precisely shaped e-beam to supply the energy to drive chemical reactions and capture their progress in real time coupled with a detailed understanding of the mechanisms by which the e-beam interacts with atoms of a sample^39–41^, enables the determination of fundamental thermodynamic parameters^15,41^ and reveals mechanisms of chemical reactions at the single-molecule level^18,42,43^. ChemTEM methodology applied to transition metals inside carbon nano test tubes provides atomic-level nanocluster dynamics analysis enabling us to compare, categorise and order this large group of transition metals with respect to their relative catalytic activity and their bonding with carbon. This study covers the whole block of early and middle transition metals consisting of three periods and five groups, which, for the first time, enables a detailed analysis of global trends within the family of transition elements. It reveals that the metal nanoclusters become more dynamic once they are engaged in reactions with carbon—one of the most unexpected outcomes of this analysis, having significant implications for understanding the atomistic workings of catalytic cycles at the nanoscale. Equally important are our findings that the tiny clusters of metals obey the Sabatier principle as heterogeneous catalysts (with three important exceptions), despite the absence of a well-defined surface, and that different metals exhibit drastically different tendencies towards defect formation in the graphenic lattice of the nanotube. This knowledge provides a new guide for the development of better catalysts for important processes involving C–C bond dissociation and formation, such as cracking and reforming that could be rationally designed and tested on the basis of ChemTEM atomic-scale experiments.

## Methods

### Materials preparation

Single-walled carbon NTs (SWNTs, Carbon solutions, USA) or double-walled carbon NTs (DWNTs, TimesNano, China) were thermally treated to open the ends of the NTs and to remove the residual amorphous carbon from the outer walls of the NTs before use. The as received NTs were heated in air for 30 min at 600 °C (SWNTs) or 570 °C (DWNTs) with a weight loss of approximately 30% observed for both samples. All other reagents and solvents were used are supplied by Sigma Aldrich.

Samples of 14 different transition metals, see Supplementary Table 1, were separately encapsulated in carbon NTs in the form of discrete, metal containing molecules, which can be easily broken down into pure metal and ligand fragments by the electron beam during HRTEM analysis.

The general method involved mixing freshly opened carbon NTs and a twofold by weight excess of the metal containing molecules. The resultant mixture was then sealed in a Pyrex glass tube under reduced pressure (10^−6^ mbar) and heated to a temperature above the sublimation point of the metal containing molecules for 3 days (Supplementary Table 1 for individual conditions). The sample was then rapidly cooled to room temperature and the NTs washed with tetrahydrofuran (100 mL) to remove any species from the outside of the nanotube and filtered through a PTFE membrane (pore size = 0.2 µm). The sample was then dispersed in isopropanol and drop cast onto a lacey carbon coated copper TEM grid for HRTEM analysis.

Formation of metal nanoclusters can also be achieved by heating the metal containing molecules inside the nanotubes to temperatures above the decomposition point of the molecules. This was achieved by sealing the nanotube sample under argon in a pyrex tube and heating at 500 °C for 3 h. Metal cluster@NT samples prepared in this way were identical to the samples formed via electron beam induced decomposition. The chemical identity of metal clusters in NTs was confirmed by the local-probe energy dispersive X-ray (EDX) analysis (Supplementary Figs. 1 and 2).

### Electron microscopy

HRTEM imaging was carried out using an image side C_s_-corrected FEI Titan 80–300 TEM operated at 80 kV acceleration voltage with a modified filament extraction voltage for information limit enhancement and an image side C_s_-corrected Zeiss Libra 200MC TEM equipped with a monochromator (0.15 eV energy slit). Images were recorded on a slow-scan CCD camera type Gatan Ultrascan XP 1000 (FEI Titan). Because a certain amount of electron dose is required to achieve atomic resolution, the exposure time for each image ranged from 0.2 to 2 s. Therefore the temporal resolution is not sufficient for atomically-resolved imaging of possible fast moving molecules, ions or atoms. For all in-situ irradiation experiments the microscopes provided a highly controllable source of local and directed electron radiation on a selected area of the sample. Experimentally applied electron-fluxes ranged from 0.9 × 10^6^ to 1.1 × 10^6^ e^−^ nm^−2^ s^−1^. For each observation, approximately 30 s were spent for adjusting the magnification, dose rate and focal length. TEM specimens were heated in air at 150 °C for 7 min shortly before insertion into the TEM column. All imaging experiments were carried out at room temperature. The representative AC-HRTEM images of different metal nanoclusters in their initial states and the corresponding fast Fourier transforms exhibiting features consistent with the nanoclusters being in a metallic state of the given metal element are shown in Supplementary Figs. 3 and 4.

EDX spectra were recorded for small bundles of SWNTs (3–10 NTs), filled with a metal (containing one or a few nanoclusters) on a JEOL 2100 F TEM equipped with an Oxford Instruments X-rays detector at 100 kV in bright-field mode (Supplementary Figs. 1 and 2).

## Electronic supplementary material

Supplementary Information

Descriptions of Additional Supplementary Files

Supplementary Movie 1

Supplementary Movie 2

Supplementary Movie 3

Supplementary Movie 4

## Data Availability

All relevant data are available from the authors.
