# Supplementary material for: Comparison of atomic scale dynamics for the middle and late transition metal nanocatalysts
Source: Nat Commun. 2018 Aug 23;9:3382. doi: 10.1038/s41467-018-05831-z (PMC6107508; doi:10.1038/s41467-018-05831-z)
Supplement: Supplementary file 1 — Supplementary Information [file 41467_2018_5831_MOESM1_ESM.pdf]

Supplementary Materials for

Comparison of Atomic Scale Dynamics for the Middle and Late Transition Metal Nanocatalysts

Kecheng Cao,[1] Thilo Zoberbier,[1] Johannes Biskupek,[1] Akos Botos,[2] Robert L. McSweeney,[2] Abdullah Kurtoglu,[2] Craig T. Stoppiello,[2] Alexander V. Markevich,[2] Elena Besley,[2] Thomas W. Chamberlain,[2,3*] Ute Kaiser,[1*] Andrei N. Khlobystov[2*]

Affiliations:

[1] Electron Microscopy of Materials Science, Central Facility for Electron Microscopy, Ulm University, Albert-Einstein-Allee 11, Ulm 89081, Germany

[2] School of Chemistry, University of Nottingham, University Park, Nottingham NG7 2RD, United Kingdom

[3] Institute of Process Research and Development, School of Chemistry, University of Leeds, Leeds LS2 9JT, United Kingdom

*Correspondence to: t.w.chamberlain@leeds.ac.uk (T.W.C); ute.kaiser@uni-ulm.de (U.K); andrei.khlobystov@nottingham.ac.uk (A.N.K.)

Supplementary Table 1 | Information about the preparation, structure, sizes and EDX analysis of the samples in our experiments.

Metal	Metal precursor	Nanotube filling conditions	Cluster structure	Cluster size (nm)
Cr	$Cr(CO)_6$	Gas phase, 120 °C for 3 days @10^{-6} mbar	Metallic	1.71 ± 0.81
Mn	$Mn(C_5HF_6O_2)_2$	Gas phase, 120 °C for 3 days @10^{-6} mbar	Metallic	1.49 ± 0.51
Fe	$Fe(C_5H_5)_2$	Gas phase, 300 °C for 3 days @10^{-6} mbar	Metallic	0.86 ± 0.28
Co	$Co(C_5H_5)_2$	Gas phase, 300 °C for 3 days @10^{-6} mbar	Metallic	1.23 ± 0.17
Ni	$Ni(C_5HF_6O_2)_2$	Gas phase, 140 °C for 3 days @10^{-6} mbar	Metallic	0.98 ± 0.11
Mo	$Mo(CO)_6$	Gas phase, 150 °C for 3 days @10^{-6} mbar	Metallic	0.90 ± 0.13
Tc	N/A			
Ru	$Ru_3(CO)_{12}$	Gas phase, 150 °C for 3 days @ 10^{-6} mbar	Metallic	1.14 ± 0.16
Rh*	$Rh_4(CO)_{12}$	Gas phase, 120 °C for 3 days @10^{-6} mbar	Metallic	1.02 ± 0.23
Pd	$Pd(C_5HF_6O_2)_2$	Gas phase, 140 °C for 3 days @10^{-6} mbar	Metallic	1.21 ± 0.40
W	$W(CO)_6$	Gas phase, 150 °C for 3 days @10^{-6} mbar	Metallic	0.95 ± 0.19
Re	$Re_2(CO)_{10}$	Gas phase, 150 °C for 3 days @10^{-6} mbar	Metallic	0.76 ± 0.10
Os	$Os_3(CO)_{12}$	Gas phase, 150 °C for 3 days @10^{-6} mbar	Metallic	1.02± 0.21

Ir*	$Ir_4(CO)_{12}$	Gas phase, 120 °C for 3 days @10^{-6} mbar	Metallic	1. 30 ± 0.41
Pt	$Pt(C_5HF_6O_2)_2$	Gas phase, 150 °C for 3 days @10^{-6} mbar	Metallic	1.03 ± 0.09

Experimental details and characterisation of the 14 transition metals in carbon nanotubes, including the metal precursor and filling conditions used for the formation of each metal cluster@nanotube sample, the resultant structure and size of the formed nanoparticles, and EDX data. *The Rh and Ir nanoclusters are confined in double-walled nanotubes as the larger size of the $Rh_4(CO)_{12}$ and $Ir_4(CO)_{12}$ precursor molecules makes them more suitable for encapsulation in the marginally wider double-walled nanotubes than SWNT. In any case, there is no obvious interaction between the metal nanoclusters and the second (outer) wall of the double-walled nanotube and, therefore, the extra, outer wall of the double-walled nanotube has no direct influence on the observed behaviour of the metal nanocluster.

The cluster sizes shown in this table are the diameters measured parallel to the NT axis. The diameters of the clusters perpendicular to the NT axis of are 1.0 ± 0.4 nm.

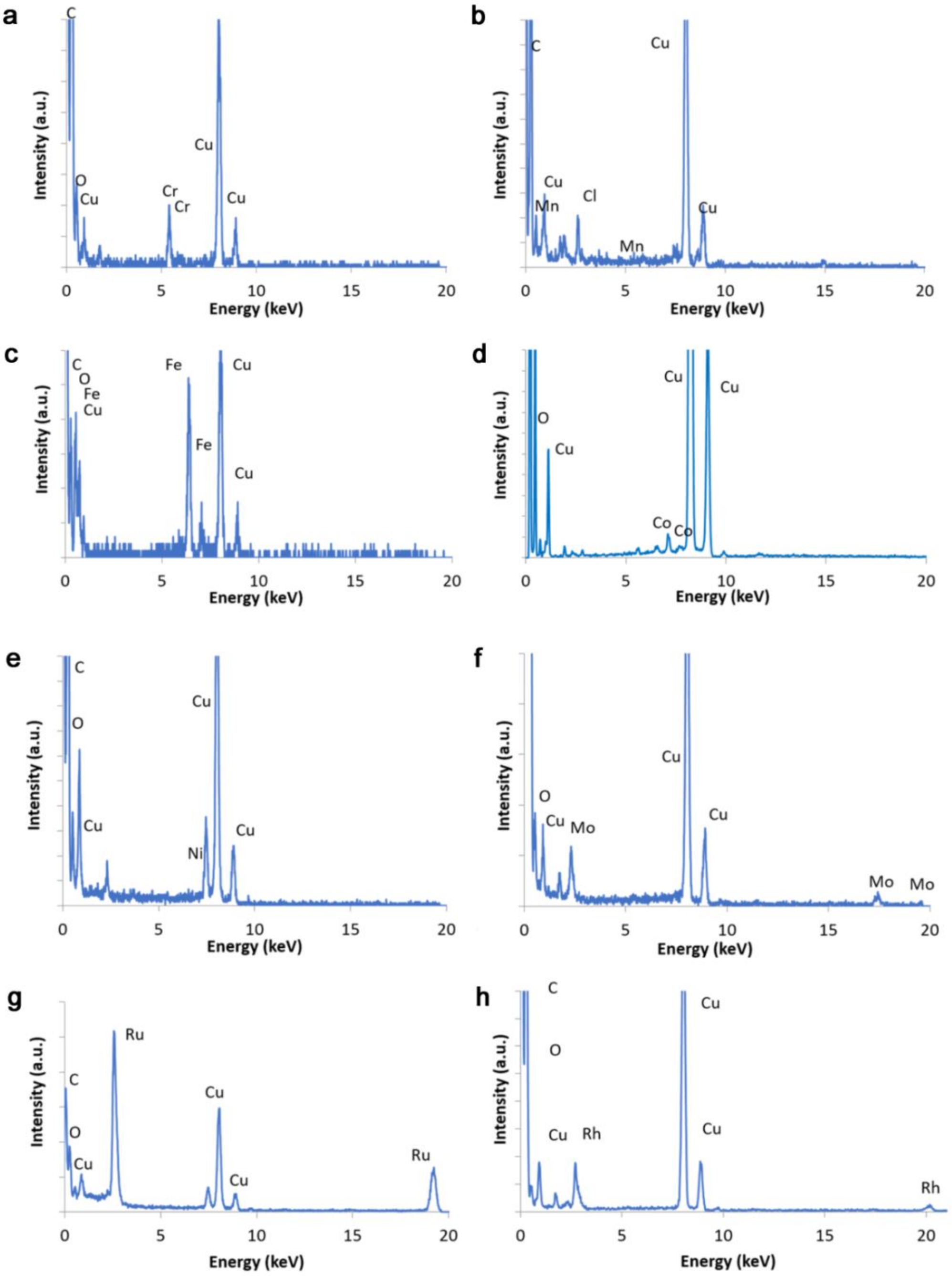

Supplementary Figure 1 | EDX spectra of (**a**) Cr, (**b**) Mn, (**c**) Fe, (**d**) Co, (**e**) Ni, (**f**) Mo, (**g**) Ru and (**h**) Rh nanoclusters embedded in carbon nanotubes recorded at 100 kV. Small bundles of SWNTs (3-10 nanotubes) filled with each metal were analysed for each sample using a narrow electron beam (5 nm diameter) illumination (N.B. Cu peaks are a result of the TEM grid).

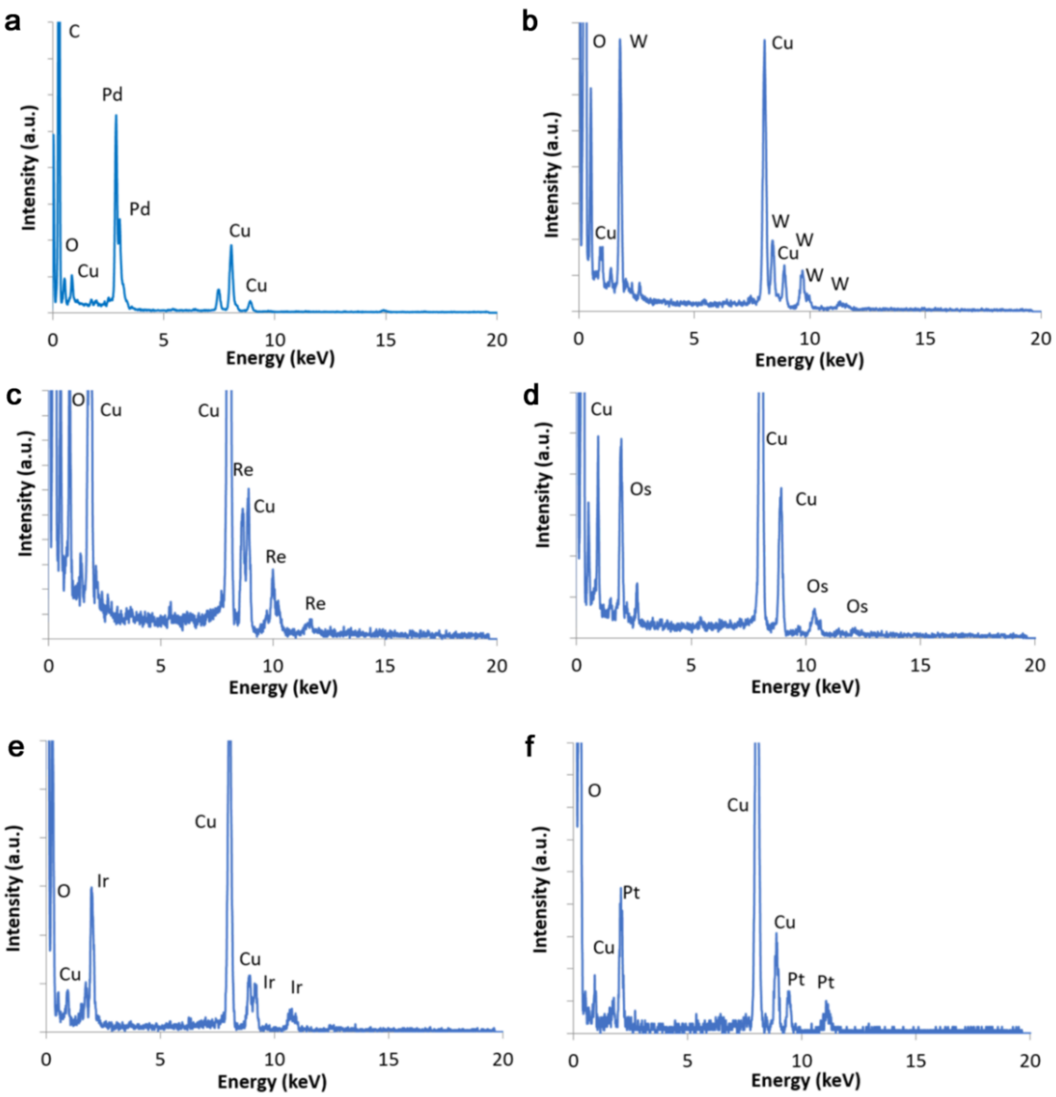

Supplementary Figure 2 | EDX spectra of (**a**) Pd, (**b**) W, (**c**) Re, (**d**) Os, (**e**) Ir and (**f**) Pt nanoclusters embedded in carbon nanotubes recorded at 100 kV. Small bundles of SWNTs (3-10 nanotubes) filled with each metal were analysed for each sample using a narrow electron beam (5 nm diameter) illumination (N.B. Cu peaks are a result of the TEM grid).

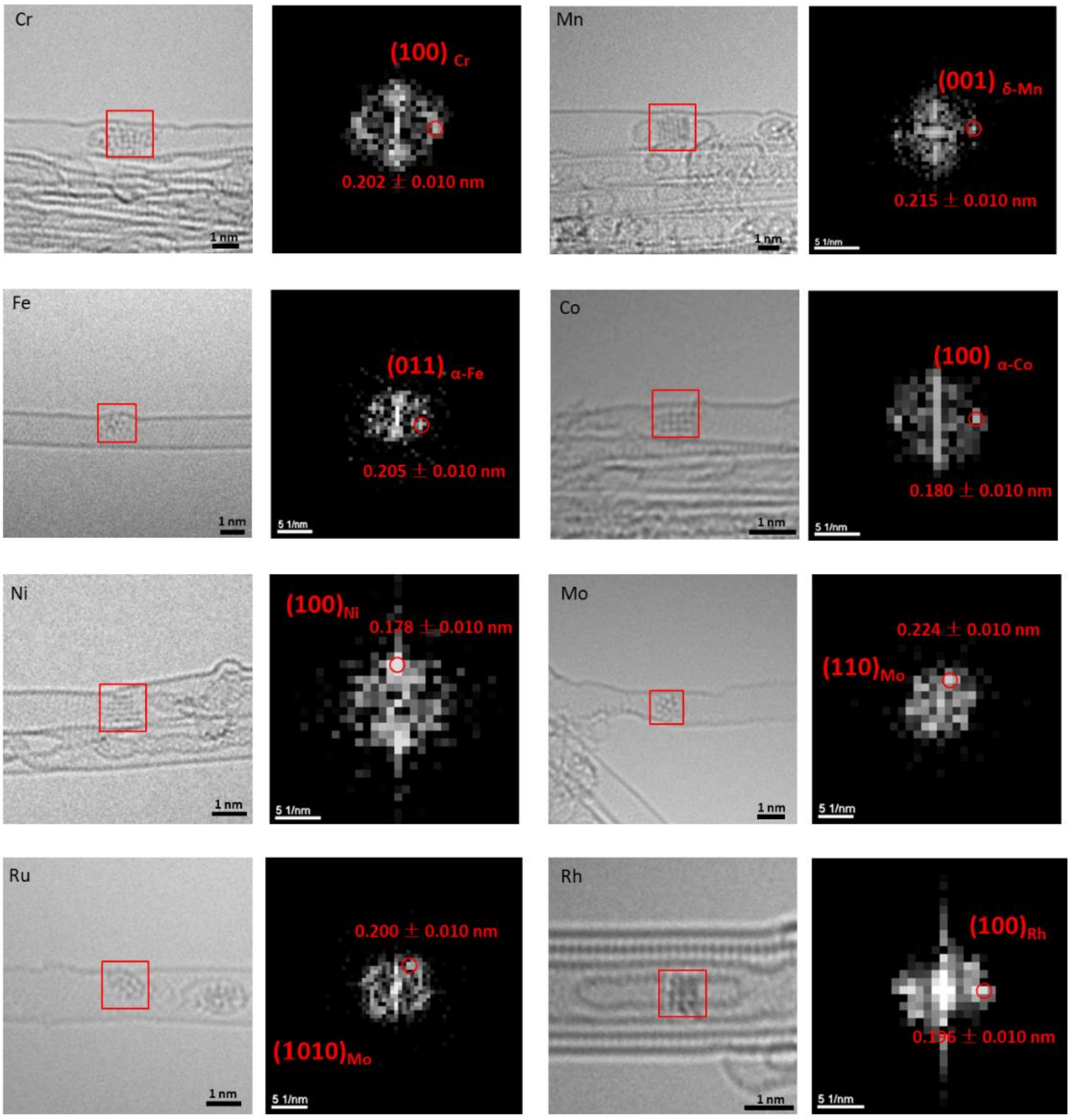

Supplementary Figure 3 | Representative AC-HRTEM images of the different metal nanoclusters in their initial states (before rearrangement induced by the e-beam has taken place), and the corresponding fast Fourier transforms (FFT) exhibiting features consistent with the nanoclusters being in a metallic state of the given metal element.

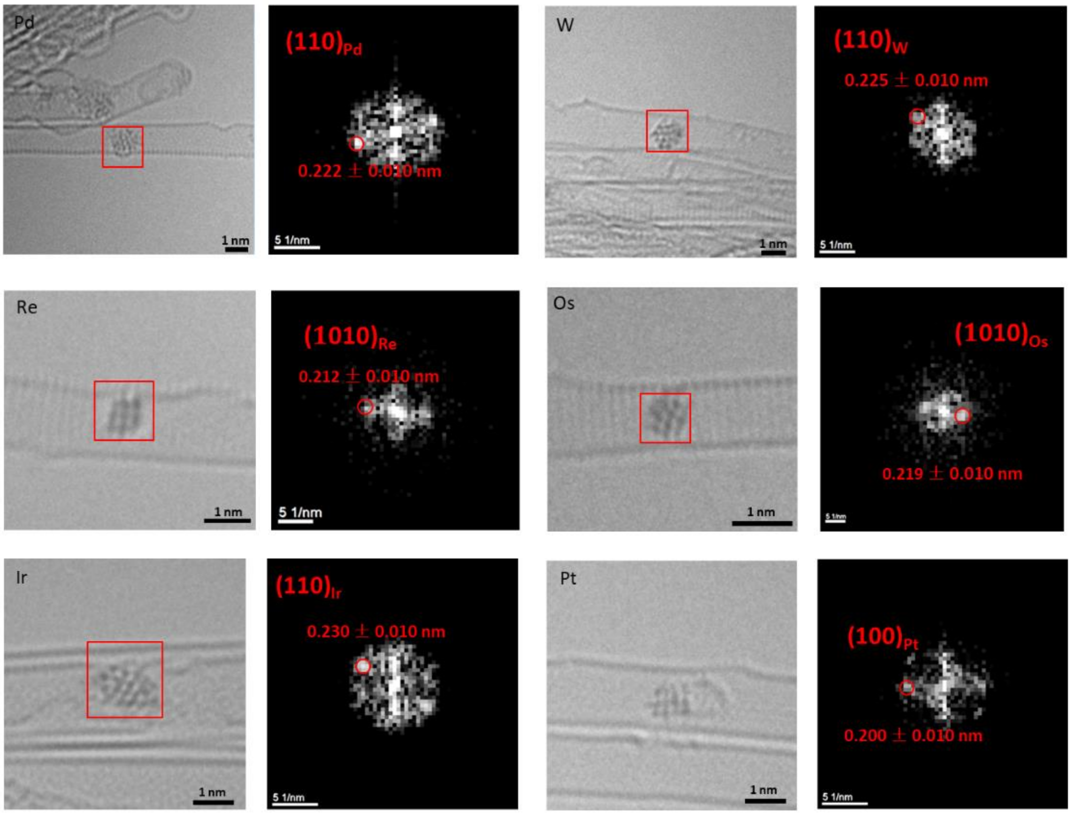

Supplementary Figure 4 | Representative AC-HRTEM images of the different metal nanoclusters in their initial states (before rearrangement induced by the e-beam has taken place), and the corresponding fast Fourier transforms (FFT) exhibiting features consistent with the nanoclusters being in a metallic state of the given metal element.

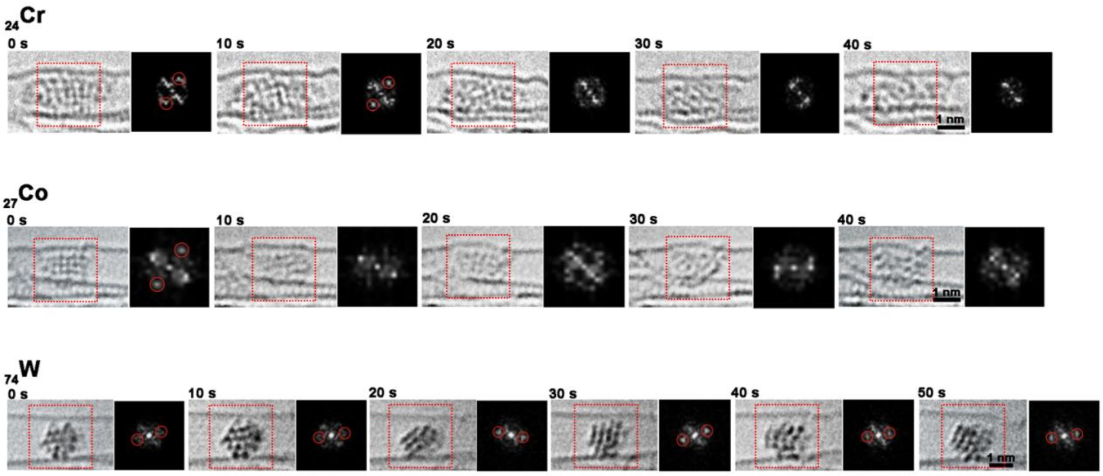

Supplementary Figure 5 | The detailed time-series AC-HRTEM images of Cr, Co and W nanocrystals with corresponding local FFT of the marked regions showing their structural transformation under 80 keV e-beam irradiation. The crystalline structures of Cr and Co nanocrystals are destroyed by the e-beam in 20 and 10 seconds respectively. In contrast, the W nanocluster maintains its crystalline features during transformations. Our in-situ investigations show this 'irradiation induced melting' process of nanocrystals at the atomic level. As shown in Fig. 2a in the main manuscript, different metals behave differently under the same conditions indicating the existence of a 'melting point' in the 'irradiation induced melting' process. The 'melting point' is determined by the chemical nature of the metal as well as a specific combination of the dose rate and the energy of e-beam.

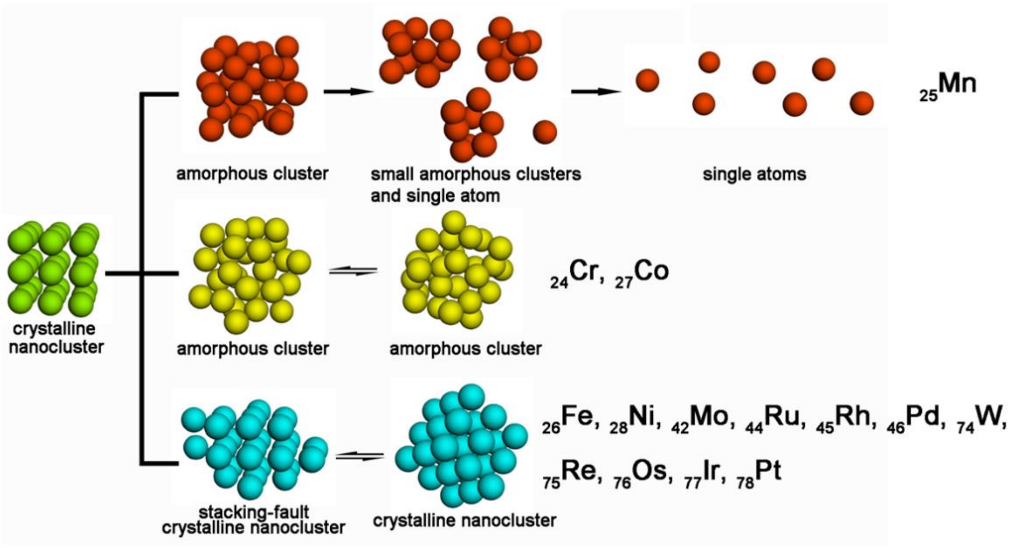

Supplementary Figure 6 | Classification of the behaviour of the 14 transition metal nanoclusters which undergo different structural transformations under the 80 keV e-beam with the same dose rate. The Mn nanocrystal is 'vaporized'. Cr and Co nanocrystals are 'melted', while other metal nanocrystals simply change their structure which retains crystalline features.

Supplementary Discussion 1. Comparing e-beam stimulated processes with thermal stimulated processes

In AC-HRTEM experiments, energy is supplied directly to the atoms, through kinetic energy transfer from the fast electrons of the e-beam to the atoms. In this respect, care should be taken in making a direct comparison with thermally activated reactions. In the section 'Dynamic behaviour of metal nanoclusters under e-beam irradiation', we note the different quasi-melt dynamic behaviour of the metal nanoclusters when irradiated by the 80 keV e-beam. For example, the Cr and Co nanocrystals are 'melted', the Mn nanocrystal is 'vaporized' and others retain crystalline features as categorised in the SI file (Supplementary Fig. 6). In Supplementary Table 2 below, we compare the melting points

reported for the 15 bulk metals, and these correlate well with the general dynamic behaviour of the metals observed in AC-HRTEM (except Cr). The actual melting points for the 1 nm nanoclusters will be several hundred degrees lower than for the bulk metal according to the Gibbs-Thompson equation[1]:

$$\Delta T_m = T_m - T_m(d) \quad = 4\sigma_{\mathrm{sl}} T_m / \left(d \Delta H_f \rho_s\right) \qquad (1)$$

where σ_{sl} is the surface energy of the solid-liquid interface, T_m is the bulk melting point, $T_m(d)$ is the melting point of crystals of size d, ΔH_f is the bulk enthalpy of fusion (per g of material), and ρ_s is the density of the solid.

For example, the calculated melting points of 1 nm nanoclusters for Mn, Fe, Co, Ni, Pd and Pt are 624.5 °C, 705.2 °C, 951.6 °C, 786.1 °C, 872.7 °C and 999.4 °C respectively. We believe that this indicates a relationship between the thermally activated processes and those activated by the e-beam in our experiments.

Supplementary Table 2 | Melting point, cohesive energy and dynamic behaviour of metals in the e-beam. Cohesive energy of the metals (E_{coh}) and maximum transferred energy (E_{T_max}) from the 80 keV e-beam for different transition metal atoms. The ratio E_{T_max}/E_{coh} represents the energy received from the e-beam as a percentage of the metal's cohesive energy.

Metal	Cr 24	Mn 25	Fe 26	Co 27	Ni 28	Mo 42	Tc 43	Ru 44	Rh 45	Pd 46	W 74	Re 75	Os 76	Ir 77	Pt 78
Bulk melting point (°C)	1907	1246	1638	1495	1455	2623	2157	2334	1964	1555	3422	3186	3033	2466	1768
E_{coh} (eV)	4.10	2.92	4.28	4.39	4.44	6.82	6.85	6.74	5.75	3.89	8.90	8.03	8.17	6.94	5.84
E_{T_max} (eV)	3.64	3.45	3.39	3.21	3.23	1.97	1.93	1.87	1.84	1.78	1.03	1.02	1.00	0.99	0.97
$[E_{T\_max}/E_{coh}] \times 100$(%)	89	118	79	73	72	29	28	28	32	46	12	13	12	14	17

Some thermally activated metal-catalysed transformations in carbon nanostructures, such as the cutting of carbon nanotubes by metal nanoparticles, can be catalysed by Ni or Co metal clusters in Ar/H_2 (9:1 in volume) at 850 °C in 30 minutes and appear to be similar to those that we observe under the e-beam[2]. Also, we show that Pt, Re, Ni, Co, Pd *etc.* nanoclusters have the ability to catalyse carbon nanotube or carbon shell formation under the e-beam, whilst Manfred R. *et al.* reported that Re catalysed the growth of NTs at 950 - 1100 °C under a CH_4 atmosphere[3]; Terrones M. *et al.* reported Co catalysed growth of NTs at 950 - 1100 °C under a triazine atmosphere[4]; Masako Y. *et al.* reported Ni catalysed growth of NTs at 600 - 1000 °C in a 2-methyl-1,2'-naphthyl ketone atmosphere[5]. From these examples it is apparent that temperatures in the range of 600 - 1100 °C are necessarily for activating transition metal catalysts for the transformation and growth of carbon structures. Therefore, we can draw parallels between the processes triggered and promoted by the 80 keV e-beam in nanotubes and analogous

processes initiated thermally at 600 - 1100 °C in bulk, even though technically the temperature of the materials in our AC-HRTEM experiments is essentially ambient, e.g. 20 - 25 ^{o}C.

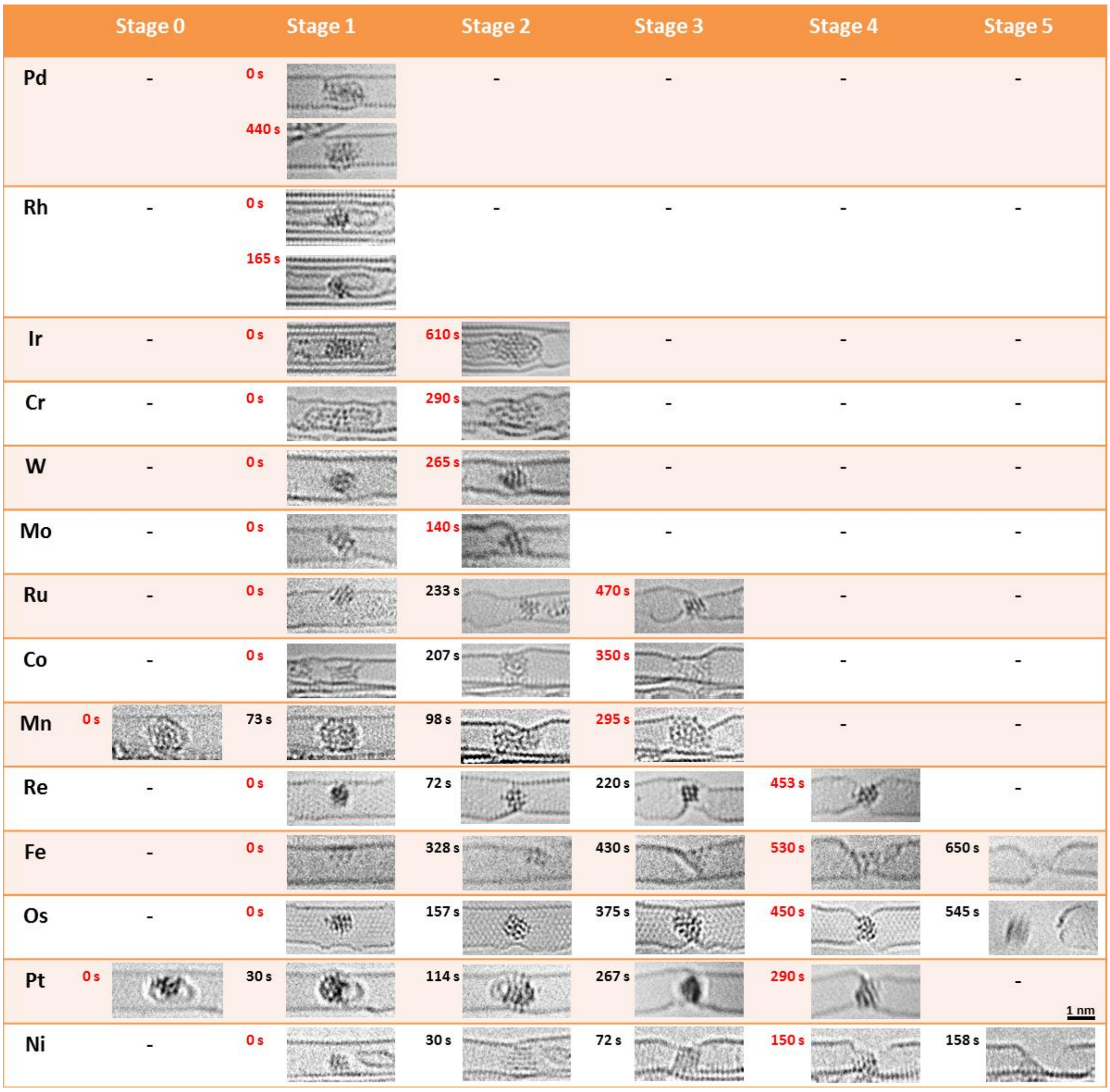

Supplementary Figure 7 | Detailed AC-HRTEM time series summarising the typical observations of the interactions and reactions of the 14 transition metal nanoclusters with carbon nanotubes corresponding to Table 2 in the main manuscript. In some cases, such as Mn or Pt, stage 1 is preceded by stage 0 which we denote as a stage in which the metal cluster is separated from the nanotube by a shell of amorphous carbon formed during materials preparation. In some cases such carbon shells were

observed to enhance the stability of the initial structure of the Mn nanocluster and inhibit interactions between the metal and nanotube (Fig. 2b). However, for Pt the effect of the carbon shell was negligible as the metal nanocluster reached stage 4 in 290 seconds thus demonstrating the highly effective nature of the Pt-C bonding with nanotube (Fig. 2b and 4b).

Supplementary Discussion 2. Reproducibility of the observed interactions

As stated in our manuscript, the specific types of metal behaviour observed in the e-beam are reproducible for a given metal from one sample area to another, provided AC-HRTEM conditions are the same (e.g. the sample is irradiated with an e-beam of the same energy and at the same dose rate). Variation in the size of the metal nanoclusters in the NTs is likely to cause some uncertainty, influencing the rate of reaction, however, the type of reaction remains largely unaffected. For example, in Supplementary Fig. 10a, a very small Re cluster (containing less than 10 atoms) catalyses transformations of a carbon structure, demonstrating the influence of nanocluster size on the rate and the extent of reaction: the small Re nanocluster catalyses the growth of a carbon nanostructure from 1.05 nm to 1.98 nm in 302 s, which is lower than in the case of a larger Re nanocluster in Fig. 4a which catalyses the growth of a carbon nanostructure from 0.11 nm to 2.29 nm in 281 s. To reiterate and expand on this, typically 50 - 700 sequential TEM images are contained in a single time series or a movie for each metal, depending on the reaction time. Our strategy is to compare and present the reactions catalysed by each type (i.e. element) of metal nanoclusters, and ascertain what the typical reaction behaviour of each metal is, thus, only behaviour of similar sized nanoclusters was compared in the study. Approximately 10 time-series were recorded for each element showing reactions,

including defect formation processes and carbon structure transformation processes, catalysed by metal nanoclusters, and between 3 - 7 time-series were acquired for each type of process observed for each element. However, the sizes of the nanoclusters in these 3 - 7 time-series do vary which make statistical analysis difficult. Therefore, our study, which may look compact at first glance, is actually based on thousands of meticulous observations.

To illustrate the reproducibility of our observations we show and compare different time-series of reactions observed for different nanoclusters of the same metal in which the nanoclusters are all similar in size to those shown in the main manuscript (Fig. 4a and b). The additional time series in Supplementary Fig. 10b illustrate the reproducibility of our experiments, showing two W nanoclusters of similar size to the W nanocluster in Fig. 4a, catalysing the growth of carbon nanostructures with a 0.45 nm length change in 307 s (Supplementary Movie 2). Similarly, the Mo nanocluster below, Supplementary Fig. 10b, catalyses the growth of a carbon nanostructure with a 0.52 nm length change in 263 s, which is similar to the catalytic reaction of Mo shown in Figure 4a. The slight variations we observe in nanocluster size do not affect our measurements of the interpretation of the observed interactions to a significant degree. Metal nanoclusters of the same element, even with slight variations in size, behave similarly during each reaction process. In this study, the size of the metal nanoclusters selected for comparison of their relative reactivities with nanotubes is approximately 1.50 ± 0.90 nm.

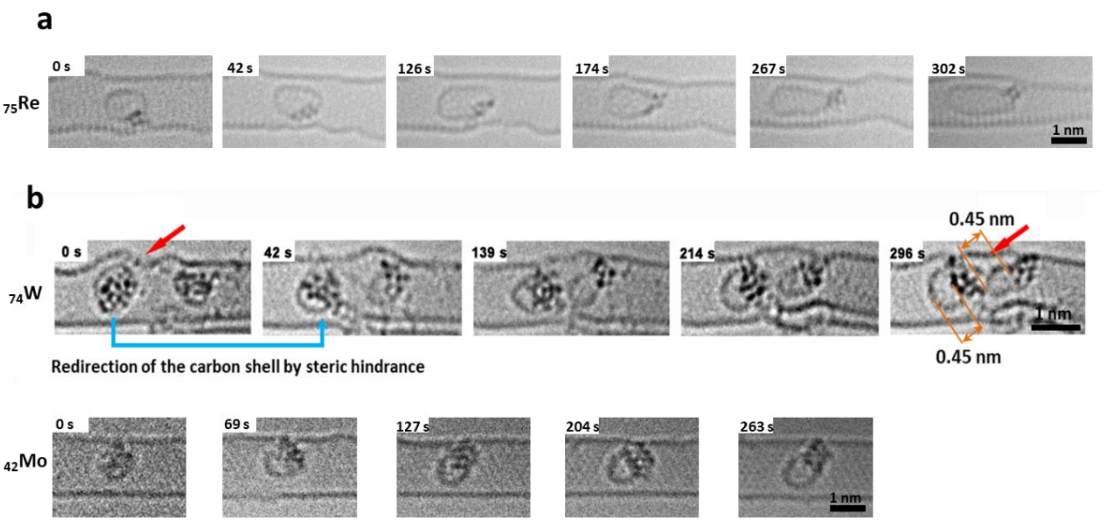

Supplementary Figure 8 | (a) Time-series in which a small Re cluster containing less than 10 atoms catalyses the growth of a carbon nanostructure under 80 keV e-beam irradiation at a slower rate than that observed for the larger nanocluster of the same metal shown in the main manuscript, demonstrating the effect of nanocluster size on rate of the reaction. (b) Two time-series for W and Mo nanoclusters respectively with similar sizes to those reported in the main paper (Fig. 4) demonstrating similar behaviour and rates of reactions, which exemplifies the high degree of reproducibility in our observations. The red arrows highlight the deformation of the host SWNT caused by the growth of the carbon nanostructures. The movie for the W nanocluster catalysed reaction is presented as Supplementary Movie 2.

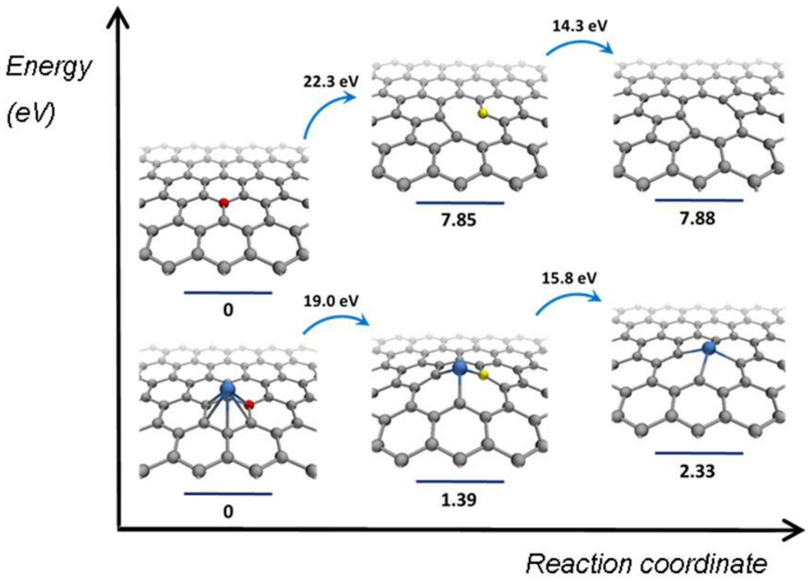

Supplementary Figure 9 | Theoretical calculations demonstrate the metal atom catalysing defect formation in the graphenic lattice due to the metal-carbon bonding. Inside the nanotube metal atoms are engaged in M-C bonding which perturbs the C-C bonds making them easier to break. Transferred energy (E_T) from the e-beam to the carbon atoms of the nanotube can provide the energy required to break the C-C bonds and eject individual carbon atoms, creating a defect within the nanotube structure which is stabilised by M-C -bonding. Theoretical calculations (the numbers above the curly arrows indicate the activation energy required from the e-beam to enable the transformation) reveal that interactions between a single metal atom (Ru) and the sp^2-hybridised carbon atoms of a graphitic lattice significantly reduce the energy threshold for carbon atom ejection from 22.3 eV for the metal-free case to 19 eV in the presence of a metal atom. The metal also significantly stabilises the mono-vacancy structure after carbon atom ejection from 7.85 eV to 1.39 eV which affects the energy threshold to ejection of carbon atoms. A further C-atom can be ejected resulting in formation of a di-vacancy defect which is similarly stabilised by the metal (c.f. formation energy of 2.33 eV compared

to 7.88 eV for the metal-free system). The momentum is transferred to the carbon atom highlighted in red and yellow for first and second impacts respectively from below, perpendicular to the graphitic lattice. The nature of the M-C interactions for different metals determines the stability of the defect and thus dictates how quickly carbon atoms are knocked out in the vicinity of the metal atom and evolve into new carbon nanostructures (e.g. protrusions, carbon shells, internal nanotubes).

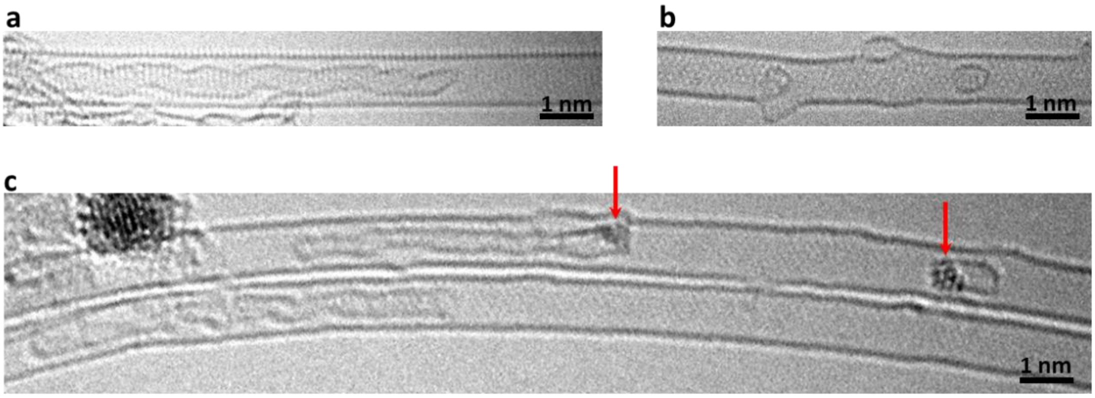

Supplementary Figure 10 | Typical AC-HRTEM images showing the native carbon nanostructures present in the NTs. During the synthesis of NTs and materials preparation, some adventitious carbon can be incorporated within the nanotube cavity which appears in the form of long undefined carbon structures (a) or small bubble-like structures (b). The native carbon structures can engage in interactions with metal nanoclusters in the early stages of AC-HRTEM observations (denoted as Stage 0 in Supplementary Fig. 7). A typical AC-HRTEM image of the Pt@NTs sample shows this kind of interaction (c). In addition, the native carbon nanostructures are notably different to the newly formed carbon nanostructures in Fig. 4, which emerge, grow and persist throughout the AC-HRTEM time-series, such as in the case of Re, Ni, Co *etc.* Mn, Fe and Cr promote the inverse processes. The connections between the metal clusters and the native carbon nanostructures enable us to observe and analyse the different behaviours and interactions between the metal clusters and carbon nanostructures.

Supplementary Table 3 | Relative rate of catalysis promoted by metal nanoclusters. Comparison of the change in total length (Δl) and surface area (ΔS) of the observed carbon structures with the number of carbon atoms added or removed compared for the 14 metal nanoclusters and considered as a measure of the individual metal's catalytic activity to promote transformations in carbon structures.

	Rh	Ir	Cr	Os	W	Mo	Ru	Pd	Co	Ni	Fe	Mn	Re	Pt
Δl (nm) (± 0.05)	0	0	- 0.10	+ 0.15	+ 0.45	+ 0.48	+ 0.57	+ 0.68	+ 0.90	+ 1.08	- 2.00	- 2.10	+ 2.18	+ 2.29
ΔS (nm^2) (± 0.10)	0	0	- 0.31	+ 0.57	+ 1.70	+ 1.96	+ 2.15	+ 2.35	+ 4.01	+ 4.75	- 5.65	- 6.28	+ 7.22	+ 7.78
Number of carbon atom (± 4)	0	0	- 12	+ 22	+ 65	+ 75	+ 82	+ 90	+ 153	+ 181	- 216	- 240	+ 276	+ 297

The one-atom-thick carbon nanostructures in Fig. 4 are hemispherical or cylindrical with a hemispherical cap in three-dimensional space. Thus, the number of carbon atoms in the carbon nanostructure is proportional to its size - the surface area. Using the formula for calculating the surface area of both a sphere and a cylinder enables the change of the surface area of the carbon nanostructure to be estimated. The atomic lattice of the one-atom-thick carbon nanostructures is likely to be graphenic, and thus the density of carbon atoms is 38.18 atoms per nm^2. This enables the number of carbon atoms added to the growing structure to be calculated as shown in Supplementary Table 3.

Supplementary References

1. Jackson, C. L. & McKenna, G. B. The melting behavior of organic materials confined in porous solids. *J. Chem. Phys.* **93,** 9002-9011 (1990)

2. Elías, A. L. et al. Longitudinal cutting of pure and doped carbon nanotubes to form graphitic nanoribbons using metal clusters as nanoscalpels. *Nano Lett.* **10**, 366-372 (2010).

3. Ritschel, M., Leonhardt A., Elefant D., Oswald S. & Büchner B. Rhenium-catalyzed growth carbon nanotubes. *J. Phys. Chem. C*, **111**, 8414-8417 (2007).

4. Terrones M. et al. Controlled production of aligned-nanotube bundles. *Nature* **388**, 52–55 (1997).

5. Yudasaka M., Kikuchi R., Matsui T., Ohki Y., & Yoshimura S. Specific conditions for Ni catalyzed carbon nanotube growth by chemical vapor deposition. *Appl. Phys. Lett.*, **67**, 2477-2479 (1995).
