# Supplementary material for: Comparison of atomic scale dynamics for the middle and late transition metal nanocatalysts
Source: Nat Commun. 2018 Aug 23;9:3382. doi: 10.1038/s41467-018-05831-z (PMC6107508; doi:10.1038/s41467-018-05831-z)
Supplement: Supplementary file 2 — Descriptions of Additional Supplementary Files [file 41467_2018_5831_MOESM2_ESM.pdf]

Descriptions of Additional Supplementary Files

File Name: Supplementary Movie 1
Description: dynamics of a Manganese nanocluster

File Name: Supplementary Movie 2
Description: dynamics of a Tungsten nanocluster

File Name: Supplementary Movie 3
Description: dynamics of a Nickel nanocluster

File Name: Supplementary Movie 4
Description: dynamics of a Platinum nanocluster
